# Investigation of the human-gut-kidney axis by fecal proteomics, highlights molecular mechanisms affected in CKD

**DOI:** 10.1016/j.heliyon.2024.e32828

**Published:** 2024-06-11

**Authors:** Sonnal Lohia, Sophie Valkenburg, Rafael Stroggilos, Vasiliki Lygirou, Manousos Makridakis, Jerome Zoidakis, Francis Verbeke, Griet Glorieux, Antonia Vlahou

**Affiliations:** a Center of Systems Biology, Biomedical Research Foundation of the Academy of Athens, 11527, Athens, Greece; b Institute for Molecular Cardiovascular Research, RWTH Aachen University Hospital, 52074, Aachen, Germany; c Department of Internal Medicine and Paediatrics, Nephrology Division, Ghent University Hospital, 9000, Gent, Belgium

**Keywords:** CKD, Fecal, Gut-kidney axis, LC-MS/MS, Proteomics

## Abstract

**Objective:**

The interplay of gut microbiota with the kidney system in chronic kidney disease (CKD), is characterized by increased concentrations of uric acid in the gut, which in turn, may increase bacterial uricase activity and may lead to the generation of uremic toxins. Nevertheless, knowledge on these underlying bidirectional molecular mechanisms is still limited.

**Methods:**

In this exploratory study, proteomic analysis was performed on fecal samples, targeting to investigate this largely unexplored biological material as a source of information reflecting the gut-kidney axis. Specifically, fecal suspension samples from patients with CKD1 (*n* = 12) and CKD4 (*n* = 17) were analysed by LC-MS/MS, using both the Human and Bacterial UniProt RefSeq reviewed databases.

**Results:**

This fecal proteomic analysis collectively identified 701 human and 1011 bacterial proteins of high confidence. Differential expression analysis (CKD4/CKD1) revealed significant changes in human proteins (*n* = 8, including proteins such as galectin-3-binding protein and prolactin-inducible protein), that were found to be associated with inflammation and CKD. The differential protein expression of pancreatic alpha-amylase further suggested plausible reduced saccharolytic fermentation in CKD4/CKD1. Significant changes in bacterial proteins (*n* = 9, such as glyceraldehyde-3-phosphate dehydrogenase and enolase), participating in various carbohydrate and metabolic pathways important for the synthesis of butyrate, in turn suggested differential butyrate synthesis in CKD4/CKD1. Further, targeted quantification of fecal pancreatic alpha-amylase and butyrate in the same fecal suspension samples, supported these hypotheses.

**Conclusion:**

Collectively, this exploratory fecal proteomic analysis highlighted changes in human and bacterial proteins reflecting inflammation and reduced saccharolytic fermentation in CKD4/CKD1, plausibly affecting the butyrate synthesis pathways in advanced stage kidney disease. Integrative multi-omics validation is planned.

## Highlights

• Fecal suspension samples from CKD1 and CKD4 patients were analysed by high resolution LC-MS/MS.
• Protein identifications were performed using both human and bacterial UniProt databases.
• Significant changes in human proteins indicated the presence of inflammation in CKD4.
• Differential *AMY2A* abundance suggest reduced saccharolytic fermentation in CKD4.
• Identified bacterial proteins indicated plausible differential butyrate synthesis in CKD4.

## Abbreviations

**ACADVL**: Very long-chain specific acyl-CoA dehydrogenase, mitochondrial
**ALPI**: Intestinal-type alkaline phosphatase
**AMY2A**: Pancreatic alpha-amylase
**AMY2B**: Alpha-amylase 2B
**araA**: l-arabinose isomerase
**BAD_1323**: UPF0210 protein BAD_1323
**BMI**: body mass index
**CKD**: chronic kidney disease
**CRP**: C-reactive protein
**eGFR**: estimated glomerular filtration rate
**ELISA**: enzyme-linked immunosorbent assay
**eno**: Enolase
**FFAR2**: free fatty acid receptor 2
**FFAR3**: free fatty acid receptor 3
**gapA**: Glyceraldehyde-3-phosphate dehydrogenase
**gcvP**: Glycine dehydrogenase (decarboxylating)
**gdhB**: NAD-specific glutamate dehydrogenase
**GO**: Gene Ontology
**GPR109A**: hydroxycarboxylic acid receptor 4
**grxA**: Glutaredoxin 1
**GSVA**: gene set variation analysis
**HDAC**: histone deacetylase
**IAA**: indole-3-acetic acid
**LC-MS/MS**: liquid chromatography-tandem mass spectrometry
**LGALS3BP**: Galectin-3-binding protein
**lp_2659**: Probable phosphoketolase 1
**LPS**: lipopolysaccharides
**pckA**: Phosphoenolpyruvate carboxykinase (ATP)
**PIP**: Prolactin-inducible protein
**PLA2**: Phospholipase A2
**PPI**: protein-protein interaction
**PTGS1**: Prostaglandin G/H synthase 1
**SCFAs**: short-chain fatty acids
**SGLT1**: Na+/glucose cotransporter 1
**ssGSEA**: single-sample gene set enrichment analysis
**UPF**: uncharacterised protein family

## 1 Introduction

The human gut microbiota corresponds to a highly complex and diverse population from approximately 1000 different species of bacteria, fungi, archaea, viruses, and unicellular eukaryotes; amounting up to a total of 3.3 million genes, approximately 250–800 times the human genome [1,2]. The human gut microbiota present in a symbiotic relationship with its host, performs complementary metabolic functions in maintaining the gut epithelial integrity and host energy homeostasis [3,4]. It is thus considered an endogenous organ [3], playing a vital role in the systemic immune response against pathogens and allergens [5] in addition to maintaining the nutritional balance [6]. It is increasingly evident that imbalances in the microbial composition (or ‘dysbiosis’), as determined by various factors including age, hygiene, nutritional intake, stress, and infectious diseases, may contribute to the development of chronic diseases, such as diabetes, cardiovascular and kidney diseases, amongst others [7]. The colonic gut microbiota is widely dominated by the bacterial phyla *Firmicutes* and *Bacteroides* (90% of bacterial population), which are, among other things, responsible for the synthesis of short-chain fatty acids (SCFAs) [8]. SCFAs are required for host energy homeostasis, restoration of intestinal epithelial tight junction protein structure, and maintenance of intestinal epithelial barrier integrity, as well as suppression of inflammation and stress-induced damage in the intestines [9,10]. In recent years, the association of SCFAs, (including butyrate, the preferred energy source for colonocytes) with the pathogenesis of kidney diseases has become apparent. Butyrate inhibits histone deacetylases (*HDAC*), reducing the negative impact of inflammatory cytokines and albuminuria [11]. Binding of butyrate to the free fatty acid receptor 2 (*FFAR2*), free fatty acid receptor 3 (*FFAR3*) and hydroxycarboxylic acid receptor 4 (*GPR109A*) has been suggested to promote kidney protection [1].

Recently, the leaky gut phenomenon has garnered significant research interest in the context of CKD. It enables the translocation of micro-organisms, uremic toxins and their precursors, and bacterial degradation products through the intestinal wall into the submucosa, stimulating leukocytes, activating innate immunity, and triggering an inflammatory response [12]. Another specific example of the gut-kidney interplay, resulting from increased uric acid concentrations, is the increase in bacterial enzymatic activity of uricase due to the exponential growth of bacterial species expressing the enzyme, including those responsible for the synthesis of uremic toxin precursors such as *Enterobacteriaceae* sp. and *Clostridium* sp. Ultimately, this shift in bacterial composition increases the production and accumulation of uremic toxins in the systemic circulation, while decreasing the production of SCFAs, which are important for maintaining the integrity of the intestinal barrier [[13], [14], [15]]. Despite recent advances, knowledge on the bidirectional interaction in the gut-kidney axis is nevertheless still limited and further molecular analyses are required for its better understanding.

In the last decade, metaproteomics has developed into an established strategy, enabling the identification and determination of the abundance of proteins originating from humans but also the gut microbiome [16], thus allowing comprehensive insights into the phenotype and ecological systems [14,17]. The identification of human and microbial proteins can potentially increase our knowledge on the host-gut axis, specifically, in the context of gut-related chronic diseases. However, as may be observed by the number of publications, despite its enormous potential, when compared to metagenomics, metaproteomics analyses are lagging behind, in part due to lack of dedicated bioinformatic solutions to perform the data analysis. In the context of CKD studies, a handful of metaproteomics investigations have been conducted, most of them involving analysis of blood plasma [18,19]. However, the blood proteome is highly complex and dynamic, with a large abundance of housekeeping proteins from all organs, and non-intestinal inflammatory proteins; making its gut-specific analysis highly challenging [20,21].

In contrast, the metaproteomic analysis of fecal samples is more promising to provide important insights into understanding the host-gut response to CKD and developing microbiome-related treatments for the disease. For instance, recently, a study combining metaproteomics and metagenomics, analysed fecal samples to understand the specific effect of *Strongyloides stercoralis* infection on early-stage CKD patients. The study reported an increase in bacterial amino-acid metabolism in early disease stages, plausibly increasing the production of uremic toxins [22]. The additional benefits of fecal samples involve their easy, non-invasive, and self-collection process; as well as the presence of proteins and inflammatory markers directly from the colon and surrounding tissues (due to leakage or exfoliation) [23]. Therefore, for the first time in the context of CKD, we performed an untargeted fecal metaproteomics analysis, using LC-MS/MS on fecal suspension samples collected from patients with CKD stage 1 (CKD1) and CKD stage 4 (CKD4), in order to study changes potentially associated with disease progression.

## 2 Materials and methods

### 2.1 Study population

Fecal samples were collected from patients with CKD stage 1 (CKD1, *n* = 12) and CKD stage 4 (CKD4, *n* = 17), recruited at the Nephrology Unit of the Gent University Hospital, Belgium, as previously described [24,25]. Characterisation of CKD stage was performed according to the calculated estimated glomerular filtration rate (eGFR) using the Chronic Kidney Disease Epidemiology Collaboration (CKD-EPI)–creatinine equation (2009), as recommended by the National Kidney Foundation Kidney Disease Outcomes Quality Initiative [26]. Written informed consent was obtained from all the 29 volunteers. The study was conducted in accordance with the Declaration of Helsinki and approved by the Medical Ethics Committee of Gent University Hospital (Ref. No. 2010/033, B67020107926). Exclusion criteria that were applied include: dialysis treatment, active infection (C-reactive protein (CRP) > 20 mg/L), immunosuppressive therapy, body mass index (BMI) > 35 kg/m^2^, inflammatory bowel disease, active malignancy, cardiovascular event in the past 3 months, pregnancy, transplantation, use of non-steroidal anti-inflammatory drugs within the past month, and age < 18 years.

### 2.2 Sample preparation

Fecal suspensions were prepared from the fecal samples as reported earlier [24,25]. Briefly, 5 mL of anaerobic phosphate buffer was added per 1 g of the feces in a 15 mL falcon tube and vortexed for 10 min. The suspensions were centrifuged at room temperature, first for 30 min at 10000×*g* followed by a second centrifugation of the supernatant for 10 min at 10000×*g*. The obtained fecal suspensions were then filtered with a 0.22 μm filter to remove viable bacteria and stored in aliquots of 500–1000 μL at −80 °C, until further processing. Samples were prepared for high-throughput proteomic analysis with the GeLC-MS protocol [27,28]. In brief, 10 μg total protein of fecal suspension, as measured by Bradford assay, were loaded on SDS-PAGE gels. Electrophoresis runs were terminated as soon as the samples entered the separating gel. The gels were fixed with 10% acetic acid, 30% methanol for 30 min, followed by three 5 min washes with water and Coomassie colloidal blue staining (overnight). After washing the excessive stain with water, protein bands from the gel were excised and cut into small pieces. De-staining of the gel pieces was carried out with a de-stain solution constituting of 40% acetonitrile, 50 mM ammonium bicarbonate, followed by reduction with 10 mM DTE in 100 mM ammonium bicarbonate solution for 20 min at room temperature. The samples were alkylated for 20 min at room temperature with 54 mM iodoacetamide in 100 mM ammonium bicarbonate solution in the dark. 100 mM ammonium bicarbonate solution, de-stain solution and ultra-pure water were used for multiple 20 min washing steps at room temperature, followed by drying using a centrifugal vacuum concentrator. Next, trypsinization was performed with the addition of 600 ng trypsin in 10 mM ammonium bicarbonate pH 8.5, per sample, followed by incubation at room temperature in the dark for 12–16 h under humidified conditions. Extraction of tryptic peptides was carried out by incubation with 50 mM ammonium bicarbonate followed by incubation with 5% formic acid, 50% acetonitrile (twice) for 15 min at room temperature with light agitation. Finally, the extracted tryptic peptides were cleaned with 0.22 μm PVDF filters, dried with a centrifugal vacuum concentrator and stored at −80 °C until LC-MS/MS analysis.

### 2.3 LC-MS/MS analysis

The LC-MS/MS analysis was performed in a similar manner as described previously [29]. The instrument used for the analysis was a Dionex Ultimate 3000 RSLC nanoflow system (Dionex, Camberley, United Kingdom) coupled with the high-resolution QExactive Orbitrap mass spectrometer (Thermo Fisher Scientific, Bremen, Germany). Each peptide sample was reconstituted in 10 μL loading solution of 0.1% v/v formic acid. At a flow rate of 5 μL/min, 5 μL of the reconstituted sample volume was loaded onto the Acclaim PepMap 100 (100 μm × 2 cm C18, 5 μm, 100 Ȧ) trapping column using the μL-Pick-Up Injection mode. For the peptide separation the Acclaim PepMapRSLC, 75 μm × 50 cm, C18, 2 μm, 100 Ȧ column (Thermo Fisher Scientific) was used for multi-step gradient elution. The temperature was maintained at 35°C for both the trap and nanoflow columns. Mobile phase A and B constituted of 2% acetonitrile: 0.1% formic acid and 80% acetonitrile: 0.1% formic acid, respectively. The peptides were eluted under a gradient starting at 2%B to 33%B at 240 min. Washing and re-equilibration of the column was performed in-between each sample injection. Proxeon nanospray ESI source in positive ion mode was used for eluent ionization, voltage of which was set at 2.6 kV and capillary temperature was maintained at 275°C. MS1 ion resolution was set to 70,000 and that for high-energy collisional dissociation at MS2 was set to 35,000. MS/MS mode scanning was set from 380 to 2000 *m*/*z*. Applying 35% collision energy of HCD, selection of top 20 multiply charged ions from each MS/MS scan was performed. The targeted precursors with 5 ppm mass tolerance were dynamically excluded for 30 s for further isolation and activation.

### 2.4 Protein identification

Raw files (.raw) obtained from the LC-MS/MS instrumental set up were analysed by Proteome Discoverer 1.4 software using the SEQUEST search engine against the entire reviewed non-redundant human (accessed on 03-01-2023; https://www.uniprot.org/> UniProtKB Keyword search: Human > Reviewed) and bacterial (accessed on 03-01-2023; https://www.uniprot.org/> UniProtKB Keyword search: Bacteria > Reviewed) databases downloaded as FASTA files. Cysteine carbamidomethylation and methionine oxidation were selected as fixed and dynamic modifications, respectively. Trypsin was selected as the digestive enzyme. During the search, a precursor mass tolerance of 10 ppm, two missed cleavage sites, peptide length of 6–144 amino acids, and 0.05 Da fragment mass tolerance were permitted, with the false discovery rate (FDR) set stringently at 0.01, yielding a high confidence output.

### 2.5 ELISA

The quantification of Pancreatic alpha-amylase (*AMY2A*; P04746) protein (56 kDa) in fecal suspension samples was performed by enzyme-linked immunosorbent assay (ELISA) using the Pancreatic Amylase Human ELISA Kit (#ab137969, Abcam, Cambridge, UK) as per the manufacturer's instructions, using 50 μL of diluted sample per well. Samples were selected for ELISA based on availability (CKD1, *n* = 10 and CKD4, *n* = 9). Preliminary ELISA runs were performed to optimize the dilution factors (range 1:2 to 1:10,000) of each fecal suspension sample. Absolute quantification values obtained from the ELISA were converted from mu/mL to ng/mL as per the manufacturer's conversion ratio of 1:4.

### 2.6 Statistical analysis

Statistical analysis was performed using the R programming software (R version 4.2.0 with IDE: R Studio Version 1.2.5, Boston, MA, USA). The precursor ion area values as defined by Proteome Discoverer 1.4, was merged using an in-house R script and were subjected to a global (per sample) normalization as per the formula: *X*′ = (*X*/sum(*Xi*)) * 10^6^; where *X* is the raw protein area; sum(*Xi*) is the sum of all raw protein areas of a given sample; and *X′* is the normalized protein area. Box and Whisker plots visualizing the distribution of total protein abundance per fecal suspension sample, as calculated by the sum of abundance of all proteins per sample were plotted to check the quality of the data. The non-parametric Mann-Whitney *U* test was utilized for defining statistical significance. The *p*-values below 0.05 were considered as statistically significant. For the visualization of the mean abundance values of statistically significant proteins in CKD1 and CKD4 groups, data were percentage scaled as per formula *Y*′ = Y*100/sum(*Yi*), where *Y* is the average-protein abundance in the respective CKD group, sum(*Yi*) is the sum of the average abundance of the protein in both CKD groups and *Y′* is the scaled abundance. All the plots presented in this manuscript were generated either in Microsoft Office Excel (version 2018) or R using the package ggplot2 (version 3.4.4). The bacterial species (from which the proteins were identified) was used as a categorical input to visualise their taxonomical hierarchy in a sunburst chart in R using the package plotme (version 0.1.0).

### 2.7 Bioinformatic analysis

For further biological insights into the proteomic profiles obtained from the human database, single-sample gene set enrichment analysis (ssGSEA) was conducted using the gene set variation analysis (GSVA) algorithm in R from the Bioconductor package (version 1.32.0) [30]. The GSVA-ssGSEA method calculates the activity scores for each pathway in each sample. Pathways used in the GSVA analysis were extracted from the MSigDB database (https://www.gsea-msigdb.org/gsea/msigdb/index.jsp, version 7.2). We specifically utilized the following collections: H – hallmark gene sets (*n* = 50), C2 – curated gene sets (*n* = 6495), C3:TFT – regulatory gene sets:all transcription factor targets (*n* = 1115), C5:GO – ontology gene sets:gene ontology (*n* = 10532), C7:IMMUNSIGDB – immunologic gene sets:ImmuneSigDB (*n* = 4872), and C8 – cell type signature gene sets (*n* = 830). The data was z-score normalized per row (per protein). Pathway activation scores were compared among the two CKD groups with the Mann-Whitney *U* test, and significance was defined at *p*-value < 0.05. Direction of over-activation for a pathway was determined with the fold difference of the means (= Mean activation score in CKD4 – Mean activation score in CKD1). Visualization of the results of the pathway activation analysis by GSVA was performed using the ComplexHeatmap package (version 1.2) in R.

For insights into the biological role of the statistically significant proteins identified from the bacterial database, Gene Ontology (GO) Term IDs were retrieved from their respective protein accession IDs using UniProtKB (https://www.uniprot.org/id-mapping). The collected GO Term IDs were inputted into the open-source online tool REVIGO (http://revigo.irb.hr/), which uses semantic similarity measures to convert long lists of GO terms to summarized molecular functions, cellular components, and biological processes. Furthermore, bacterial species generating 10 or more proteins were short-listed, and the top four species were determined by the highest number of identified proteins originating from the respective bacterial species. The protein interaction network and pathway enrichment analysis for proteins mapped to the top four bacterial species, were performed using the protein-protein interaction (PPI) networks, STRING database (https://string-db.org/, version 11) at the default settings [31].

## 3 Results

### 3.1 Study design

An exploratory untargeted fecal metaproteomic analysis was carried out, targeting to investigate proteomic alterations in the context of the human-gut-kidney axis in CKD. As depicted in Fig. 1, fecal samples from CKD1 (*n* = 12) and CKD4 (*n* = 17) patients were collected and stored as fecal suspensions, which were then prepared for proteomic analysis according to the GeLC-MS protocol [27,28], followed by high-resolution LC-MS/MS. Proteins of both human and bacterial origin were identified, followed by extensive statistical and bioinformatic analysis to obtain biological insights.

**Fig. 1:**
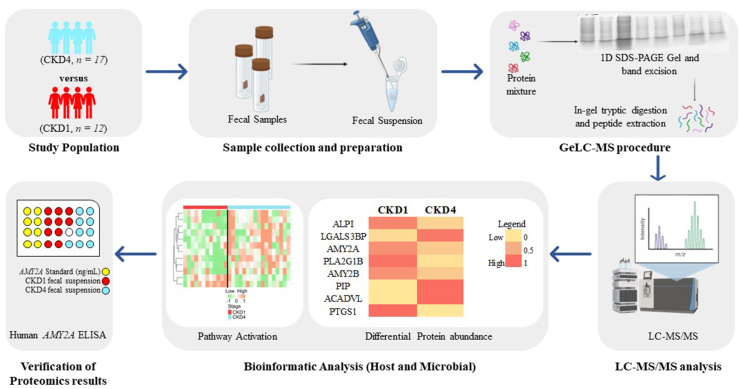
**Study design**. Fecal suspension samples from patients with CKD1 (*n* = 12) and CKD4 (*n* = 17) were processed using the GeLC-MS protocol, followed by high resolution LC-MS/MS. Functional annotation and pathway analysis were performed on both human and bacterial protein identifications, followed by ELISA for verification of proteomics results. CKD: chronic kidney disease; LC-MS/MS: liquid chromatography-tandem mass spectrometry; ALPI: Intestinal-type alkaline phosphatase; LGALS3BP: Galectin-3-binding protein; AMY2A: Pancreatic alpha-amylase; PLA2: Phospholipase A2; AMY2B: Alpha-amylase 2B; PIP: Prolactin-inducible protein; ACADVL: Very long-chain specific acyl-CoA dehydrogenase, mitochondrial; PTGS1: Prostaglandin G/H synthase 1.

Characteristics of the twenty-nine patients participating in this study are provided in Table 1. Detailed information on the cause of kidney failure per patient is further provided in Table S1.

**Table 1:** Patient characteristics (mean ± SD are provided). BMI: body mass index; CKD: chronic kidney disease; eGFR: estimated glomerular filtration rate; CRP: C-reactive protein.

Clinical parameters	CKD1	CKD4	*p*-value
	(*n* = 12)	(*n* = 17)	
Age (years)	44.17 ± 14.33	73.06 ± 10.25	<0.05
Sex (% Females)	41.67	23.53	0.332
BMI (kg/m^2^)	26.40 ± 2.54	27.40 ± 3.53	0.467
eGFR (1.73 m^2^/ml/min)	113.31 ± 14.40	23.34 ± 4.48	<0.05
CRP (mg/L)	5.65 ± 12.94	5.19 ± 7.42	0.922
Serum Urea (mg/dL)	28.83 ± 7.22	100 ± 30.59	<0.05
Cholesterol (mg/dL)	168.75 ± 31.58	192.3 ± 53.16	0.288
Serum creatinine (mg/dL)	0.75 ± 0.12	2.58 ± 0.63	<0.05
Urinary creatinine (mg/dL)	82.17 ± 53.12	69.06 ± 35.42	0.503
Diabetes (*n*)	0	5	–
Cause of kidney failure known (*n*)	7	13	–
Fecal butyrate (μmol/g feces)	15.65 ± 8.84	12.01 ± 8.59	0.33

### 3.2 Human proteins in the fecal samples

MS data analysis from all the 29 fecal suspension samples, yielded a total of 701 proteins of human origin. An average of 202 ± 48 proteins and 237 ± 90 proteins per fecal suspension sample of the CKD1 and CKD4 groups, respectively, were identified. Detailed information for all 701 protein identifications is provided in Table S2. A quality check of the data was performed based on the distribution of total protein abundance per sample, as depicted in Fig. 2a; as shown, consistency amongst the different samples was observed. Statistical assessment of the protein abundance in CKD4 versus CKD1, revealed eight significantly different (*p*-value <0.05) proteins of human origin. The distribution of average (*n* = 29) protein abundance of these proteins amongst the 701 (in total) identified proteins was also examined, indicating that they span the detected abundance range (Fig. 2b; red spots).

**Fig. 2:**
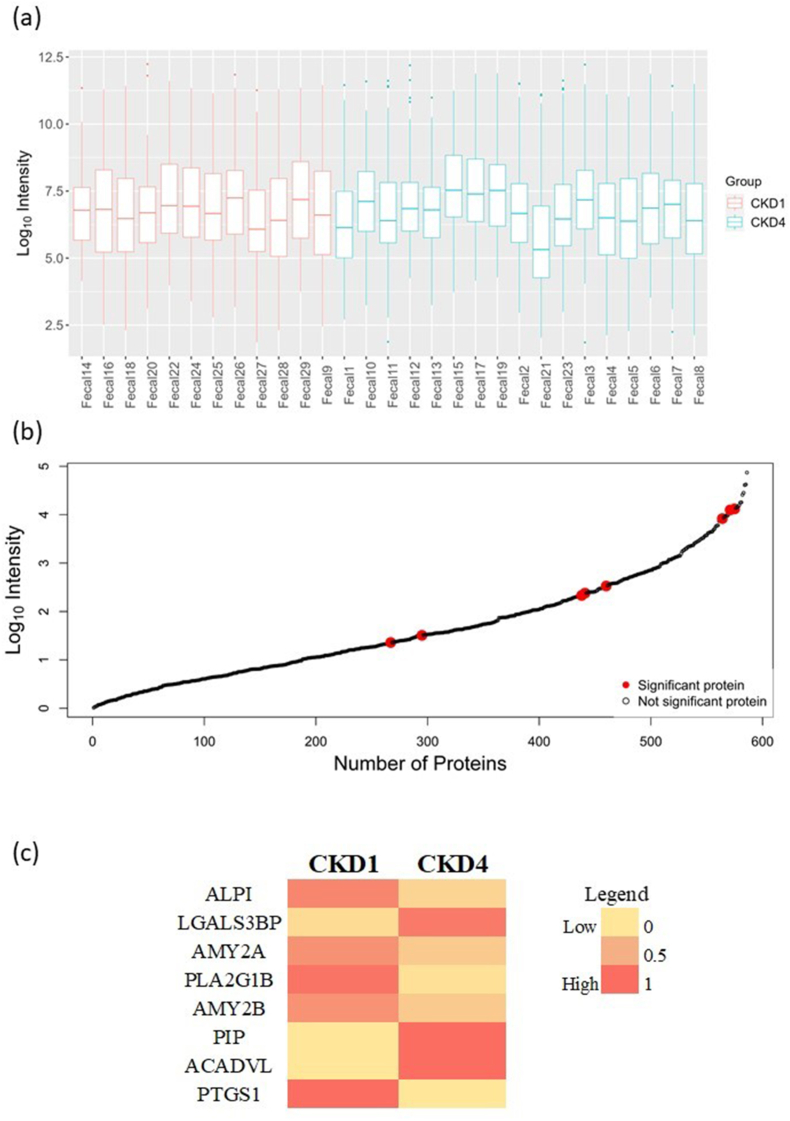
**Human proteins in the fecal samples**. (a) Distribution of total protein abundance per fecal suspension sample (b) Distribution of protein abundance for all 701 proteins of human origin; red dots indicate the statistically significant proteins (Mann-Whitney *U* test, *p*-value < 0.05) for CKD4/CKD1 (c) Heatmap visualizing the percentage-scaled mean abundance of the statistically significant proteins in the two CKD groups. CKD: chronic kidney disease; ALPI: Intestinal-type alkaline phosphatase; LGALS3BP: Galectin-3-binding protein; AMY2A: Pancreatic alpha-amylase; PLA2: Phospholipase A2; AMY2B: Alpha-amylase 2B; PIP: Prolactin-inducible protein; ACADVL: Very long-chain specific acyl-CoA dehydrogenase, mitochondrial; PTGS1: Prostaglandin G/H synthase 1. (For interpretation of the references to colour in this figure legend, the reader is referred to the Web version of this article.)

The eight statistically significant and differentially abundant proteins (respective heatmap shown in Fig. 2c) are listed in Table 2. Six out of these eight proteins were enzymes, namely Intestinal-type alkaline phosphatase (P09923; *ALPI*; down-regulated in CKD4), Pancreatic alpha-amylase (P04746; *AMY2A*; down-regulated in CKD4), Phospholipase A2 (P04054; *PLA2G1B*; down-regulated in CKD4), Alpha-amylase 2B (P19961; *AMY2B*; down-regulated in CKD4), Very long-chain specific acyl-CoA dehydrogenase, mitochondrial (P49748; *ACADVL*; identified only in CKD4) and Prostaglandin G/H synthase 1 (P23219; *PTGS1*; identified only in CKD1), considered to originate mainly from the pancreas, liver, and kidneys. Two proteins, namely Galectin-3-binding protein (Q08380; *LGALS3BP*; up-regulated in CKD4) and Prolactin-inducible protein (P12273; *PIP*; identified only in CKD4) are predominantly associated with exocrine glands.

**Table 2:** Differentially abundant proteins (CKD4/CKD1) identified from the human database.

UniProt ID	Protein name	Gene Symbol	Frequency		Average abundance (ppm)		*p*-value	Fold Change	Log2FC	Function
			**CKD1 (*n* = 12)**	**CKD4 (*n* = 17)**	**CKD1**	**CKD4**		**(CKD4/CKD1)**	**(CKD4/CKD1)**	*Source: UniProt*
P09923	Intestinal-type alkaline phosphatase	*ALPI*	12	14	15206	3404	0.002	0.22	−2.16	Hydrolysis of various phosphate compounds.
Q08380	Galectin-3-binding protein	*LGALS3BP*	3	12	60	531	0.007	8.75	3.13	Promotes integrin-mediated cell adhesion.
P04746	Pancreatic alpha-amylase	*AMY2A*	12	11	20942	7714	0.009	0.37	−1.44	Catalyzes digestion of starch and maintains glucose homeostasis.
P04054	Phospholipase A2	*PLA2*	5	2	480	27	0.039	0.06	−4.14	Has anti-helminth activity in a process regulated by gut microbiota.
P19961	Alpha-amylase 2B	*AMY2B*	8	4	19692	7540	0.045	0.38	−1.38	Catalyzes digestion of starch and glycogen.
P12273	Prolactin-inducible protein	*PIP*	0	5	0	54	0.047	Identified only in CKD4	Identified only in CKD4	Possible uremic toxin.
P49748	Very long-chain specific acyl-CoA dehydrogenase, mitochondrial	*ACADVL*	0	5	0	38	0.047	Identified only in CKD4	Identified only in CKD4	Breakdown of fatty acids into acetyl-CoA, to produce energy from fats.
P23219	Prostaglandin G/H synthase 1	*PTGS1*	3	0	577	0	0.036	Identified only in CKD1	Identified only in CKD1	Key enzyme in generation of prostaglandins; plays an important role in cyto-protection.

**Fig. 3:**
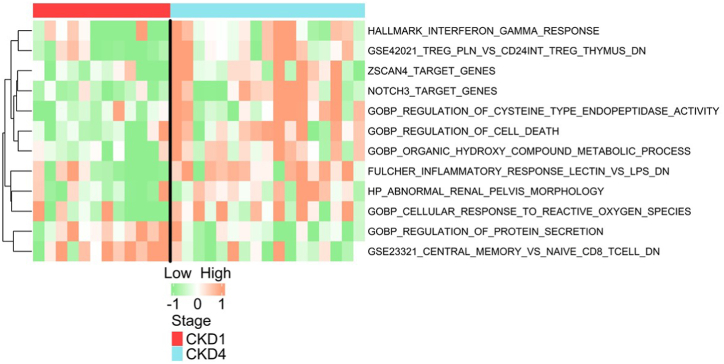
**Pathway analysis for fecal human proteins by GSVA**. Activation scores for 12 statistically significant pathways represented by the fecal (human) proteins, relevant to the gut-kidney-axis are depicted in a heatmap. The orange nodes represent elevated activation scores in the respective CKD group. CKD: chronic kidney disease; DN: down-regulation. (For interpretation of the references to colour in this figure legend, the reader is referred to the Web version of this article.)

The gene names of the 701 proteins identified from the human database, were further used as an input for pathway activation analysis with the GSVA-ssGSEA algorithm. In total, 2853 pathways could be linked to the input genes, out of which a total of 136 pathways had significantly altered activation scores between the two CKD groups (*p*-value <0.05; detailed statistical output is provided in Table S3). Fig. 3 shows a heatmap with the sample-specific activation scores, for 12 out of the 136 significantly altered pathways (selection was based on relevance and importance in CKD) in the CKD4 group versus CKD1 group comparison. Most of the significant pathways were associated with energy metabolism (example: GOBP_ORGANIC_HYDROXY_COMPOUND_METABOLIC_PROCESS; significantly activated in CKD4 and GOBP_REGULATION_OF_PROTEIN_SYNTHESIS; significantly activated in CKD1). In addition, pathways predicted to be significantly activated in the CKD4 group in comparison to CKD1 group were associated to cell death/toxicity (example: GOBP_REGULATION_OF_CELL_DEATH and GSE42021_TREG_PLN_VS_CD24INT_TREG_THYMUS_DN), damage to the kidneys (example: HP_ABNORMAL_RENAL_PELVIS_MORPHOLOGY) inflammation (example: FULCHER_INFLAMMATORY_RESPONSE_LECTIN_VS_LPS_DN), cell differentiation (example: GOBP_CELLULAR_RESPONSE_TO_REACTIVE_OXYGEN_SPECIES) and immune system response (example: HALLMARK_INTERFERON_GAMMA_RESPONSE). Pathways significantly elevated in the CKD1 group in comparison to CKD4 group could be associated with reduced cell toxicity (example: GSE23321_CENTRAL_MEMORY_VS_NAIVE_CD8_TCELL_DN).

### 3.3 Decreasing trend of AMY2A levels verified by ELISA

**Fig. 4:**
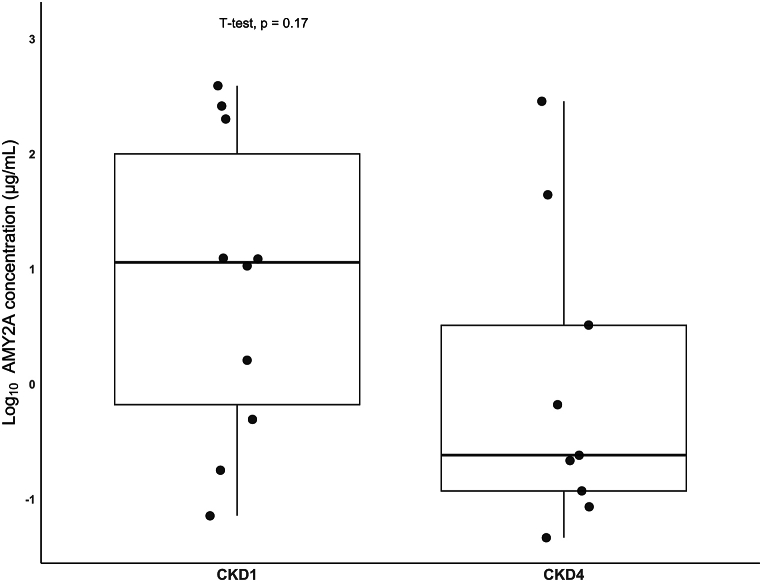
**Decreasing trend of AMY2A levels verified by ELISA**. AMY2A protein levels in fecal suspension samples of patients with CKD1 (*n* = 10) and CKD4 (*n* = 9) as quantified by ELISA.

The results of the proteomic analysis, predicting the significant down-regulation of human proteins *AMY2A* and *AMY2B* in CKD4 versus CKD1; were considered of special interest. Collectively, the two digestive enzymes were identified at high abundance and frequency in the fecal suspension samples (listed in the top 10 most abundant proteins of human origin). However, no studies directly linking these digestive enzymes to inflammation and/or CKD could be found in the literature. We hypothesized that the down-regulation of these proteins in advanced CKD may be reflective of an imbalance in the glucose homeostasis and reduced starch digestion, i.e., reduced saccharolytic fermentation, important for the synthesis of SCFAs such as butyrate. To explore this hypothesis, a pilot study was conducted, targeting quantification of the *AMY2A* protein. Of the same fecal suspension samples analysed by mass spectrometry, 10 CKD1 and 9 CKD4 samples, as per availability, were further analysed by ELISA to quantify *AMY2A* levels. Fig. 4 illustrates a decreasing trend in *AMY2A* concentration from 89.37 ± 13.55 μg/mL in the CKD1 group to 37.40 ± 89.53 μg/mL in the CKD4 group, with a fold change (CKD4/CKD1) of 0.42, in line to the proteomics results. Nevertheless, no statistical significance could be reached even after correction for moisture content of the samples, likely due to the large inter-variability of *AMY2A* protein abundance (range 0.05–391.99 μg/mL) and the small sample size (ELISA data in detail, along with the standard curve are provided in Table S4).

### 3.4 Bacterial proteins in the fecal samples

**Fig. 5:**
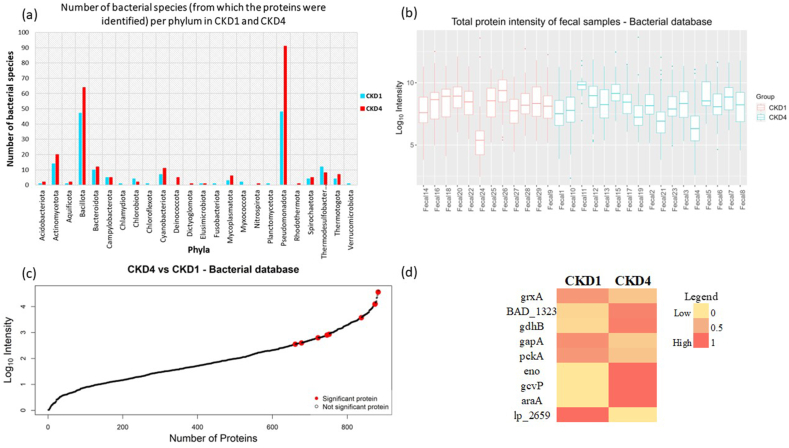
**Bacterial proteins in the fecal samples** (a) Number of bacterial species per phylum originating from the CKD1 and CKD4 group. (b) Distribution of total protein abundance per fecal suspension sample (c) Distribution of protein abundance for all the 1011 proteins identified from the bacterial database; red dots indicate the statistically significant proteins (d) Heatmap visualizing the percentage-scaled mean abundance of the statistically significant proteins in the two CKD groups. CKD: chronic kidney disease; grxA: Glutaredoxin 1; BAD_1323: UPF0210 protein BAD_1323; gdhB: NAD-specific glutamate dehydrogenase; gapA: Glyceraldehyde-3-phosphate dehydrogenase; pckA: Phosphoenolpyruvate carboxykinase (ATP); eno: Enolase; gcvP: Glycine dehydrogenase (decarboxylating); araA: l-arabinose isomerase; lp_2659: Probable phosphoketolase 1. (For interpretation of the references to colour in this figure legend, the reader is referred to the Web version of this article.)

In parallel to the analysis of human proteins, the fecal samples were also investigated for the presence of bacterial-origin proteins. In the search with the bacterial database, a total of 3058 proteins were identified from the 29 fecal suspension samples. After excluding the duplicates (*n* = 1721; protein entries that were repeated due to identification in more than one fecal suspension sample), proteins of human origin (*n* = 281) and additional contaminants (*n* = 45 proteins of non-bacterial origin including sheep, fish, fungi, amoeba amongst others present in the downloaded UniProtKB database FASTA), a total of manually cross-checked, 1011 proteins of bacterial origin, were identified from the 29 fecal suspension samples (Table S5). These proteins originated from a total of 394 bacterial species and the distribution of number of bacterial species per phylum identified in CKD1 and CKD4 groups, is represented in Fig. 5a. Furthermore, the taxonomical hierarchy of all the 394 bacterial species identified from the CKD1 and CKD4 groups are represented as sunburst charts, respectively, in the Supplementary figures (CKD1_Taxa and CKD4_Taxa).

On average, 88 ± 39 proteins, and 118 ± 88 proteins per fecal suspension sample of the CKD1 and CKD4 groups, respectively, were identified from the bacterial database. The large variation in the number of proteins identified per sample is in line with existing evidence, supporting that the human microbiome varies drastically between individuals depending on genetic, lifestyle and environmental factors [15,[32], [33], [34]], also depicted in Fig. 5b. In addition, variations were observed in the number of proteins identified per species ranging from 10 (*Clostridium botulinum*, *Clostridium perfringens*, *Parabacteroides distasonis* and *Salmonella typhimurium*) up to 172 (*Escherichia coli*). The four bacterial species generating the highest number of proteins in the analysis were *Bacteroides fragilis* (45 proteins), *Bacteroides thetaiotaomicron* (30 proteins), *Phocaeicola vulgatus* (33 proteins) and *Escherichia coli* (172 proteins). Pathway enrichment and protein-protein interaction network analysis using as input all proteins detected from these four species, commonly suggested the participation of microbiome in the synthesis, metabolism, and degradation of carbohydrates and amino acids, important for food digestion and energy production in the host (Table S6). Following statistical analysis, the abundance of the nine proteins of bacterial origin was significantly different (*p*-value < 0.05) in CKD4 compared to CKD1. The distribution of the average protein abundance of these proteins amongst the 1011 proteins, highlights their relatively high abundance amongst the detected proteins (Fig. 5c; red spots).

The nine statistically differentially abundant proteins of bacterial origin (heatmap shown in Fig. 5d; listed in Table 3) included two enzymes, NAD-specific glutamate dehydrogenase (P94316; *gdhB*; up-regulated in CKD4) and Glycine dehydrogenase (decarboxylating) (Q5LDN2; *gcvP*; identified only in CKD4) that were identified from the bacterial species *Bacteroides fragilis*, which is considered to be involved in butyrate synthesis [8]. In addition, five other enzymes were statistically differentially abundant in CKD4 vs CKD1 groups, namely Glyceraldehyde-3-phosphate dehydrogenase (B7LQ20; *gapA*; down-regulated in CKD4) identified from *Escherichia fergusonii*, Phosphoenolpyruvate carboxykinase (ATP) (C4Z0Q6; *pckA*; down-regulated in CKD4) from *Lachnospira eligens*, Enolase (B2RLL7; *eno*; identified only in CKD4) from *Porphyromonas gingivalis*, l-arabinose isomerase (Q03PR5; *araA*; identified only in CKD4) from *Levilactobacillus brevis* and Probable phosphoketolase 1 (Q88U67; *lp_2659*; identified only in CKD1) from *Lactiplantibacillus plantarum*; all of which play essential roles in carbohydrate degradation and biosynthesis pathways including glycolysis, gluconeogenesis and pentose phosphate pathways. Two additional proteins, Glutaredoxin 1 (P68688; *grxA*; down-regulated in CKD4) identified from *Escherichia coli* and UPF0210 protein BAD_1323 (A1A321; *BAD_1323*; up-regulated in CKD4) identified from *Bifidobacterium adolescentis*, were also statistically significant when comparing the CKD4 with the CKD1 group. The former protein (*grxA*) functions as an electron carrier in the glutathione-dependent synthesis of deoxyribonucleotides while the function of the later protein (uncharacterised protein family, UPF) is unknown. Results from the Gene Ontology (GO) analysis (Table S7) revealed that the identified fecal bacterial proteins are predominantly located in the cytoplasm (which could include both secreted proteins and proteins from cell lysis) and to a lesser extent in cell surface and extracellular region. They are mainly involved in processes associated with metabolism of carbohydrate and amino acids, which are crucial to produce energy in the form of NADP and ATP. The activation of cellular response to oxidative stress was also predicted to be represented by the detected differentially abundant bacterial proteins (Table S7).

**Table 3:** Differentially abundant proteins (CKD4/CKD1) identified from the bacterial database.

UniProt ID	Protein name, *Bacterial species*	Gene Symbol	Frequency		Average abundance (ppm)		*p*- value	Fold change	Log2FC	Function
			**CKD1 (*n* = 12)**	**CKD4 (*n* = 17)**	**CKD1**	**CKD4**		**(CKD4/CKD1)**	**(CKD4/CKD1)**	*Source: UniProt*
P68688	Glutaredoxin 1, *Escherichia coli*	*grxA*	10	5	1175	529	0.010	0.45	−1.15	Removes protein glutathionylation.
A1A321	UPF0210 protein BAD_1323, *Bifidobacterium adolescentis*	*BAD_1323*	3	11	1118	5602	0.021	5.01	2.32	–
P94316	NAD-specific glutamate dehydrogenase, *Bacteroides fragilis*	*gdhB*	2	10	3001	18995	0.027	6.33	2.66	Possible function: Synthesis of ammonia/urea & Succinate.
B7LQ20	Glyceraldehyde-3-phosphate dehydrogenase, *Escherichia fergusonii*	*gapA*	10	7	59287	20432	0.033	0.35	−1.54	Glycolysis.
C4Z0Q6	Phosphoenolpyruvate carboxykinase (ATP), *Lachnospira eligens*	*pckA*	12	13	18552	8559	0.049	0.46	−1.12	Gluconeogenesis.
B2RLL7	Enolase, *Porphyromonas gingivalis*	*eno*	0	8	0	1075	0.008	Identified only in CKD4	Identified only in CKD4	Glycolysis, Gluconeogenesis.
Q5LDN2	Glycine dehydrogenase (decarboxylating), *Bacteroides fragilis*	*gcvP*	0	6	0	680	0.026	Identified only in CKD4	Identified only in CKD4	Glyoxylate and dicarboxylate to 2-oxoglutarate.
Q03PR5	l-arabinose isomerase, *Levilactobacillus brevis*	*araA*	0	5	0	604	0.047	Identified only in CKD4	Identified only in CKD4	Pentose phosphate pathway.
Q88U67	Probable phosphoketolase 1, *Lactiplantibacillus plantarum*	*lp_2659*	3	0	2070	0	0.036	Identified only in CKD1	Identified only in CKD1	Pentose phosphate pathway, Methane metabolism and Carbon fixation.

### 3.5 Expression of proteins involved in butyrate synthesis

In addition to the statistically significant differentially abundant bacterial proteins, 32 bacterial enzymes directly catalyzing essential steps in the butyrate synthesis pathways were also identified in this study. Three of the 32 proteins detected in both CKD groups were Butyryl-CoA:acetate CoA-transferase identified from *Anaerostipes caccae* and *Roseburia hominis*; Acetate kinase from *Finegoldia magna*, *Clostridium botulinum* and *Phocaeicola vulgatus*; and Formate acetyltransferase from *Clostridium pasteurianum*. Out of the other 29 enzymes representing butyrate synthesis pathways, 7 and 22 proteins were identified only in one sample of the CKD1 and CKD4 groups, respectively (Table S8).

## 4 Discussion

This exploratory study was set out to increase comprehensiveness, on a molecular level, of the underlying bidirectional interplay between the gut microbiota and the kidneys in CKD, by proteomics analysis of fecal samples from patients with CKD. This largely under-explored biological material is very likely an important source of relevant information, reflecting the gut-kidney axis at the protein level. Specifically, metaproteomic analysis of fecal suspension samples from patients with CKD1 (*n* = 12) and CKD4 (*n* = 17) was performed. Our study identified the fecal proteome to comprise of almost 40% human (*n* = 701) and 60% bacterial (*n* = 1011) proteins, highlighting that the fecal proteome can be used to study the gut microbiota as a proxy, and consequently the gut-kidney axis. These results are in line with previously published metaproteomics approaches on fecal samples, reporting similar numbers and ratio of human/bacterial proteins, as in our study [35,36].

Interestingly, the fecal proteome, as studied via our metaproteomic approach, predominantly reflected molecular pathways associated with carbohydrate metabolism. This finding is in agreement with previous results from Erickson *et al*. [37], exploring the human host-microbiota signature of Crohn's disease by an integrated metagenomic and metaproteomic approach, performed on six pairs of twins [37]. Human proteins segregating into pathways related to response to reactive oxygen species, bacterial metabolites and lipid metabolism were significantly activated in CKD4 in comparison to CKD1. Similarly, multiple pathways associated to cell death, as well as reflecting CD4 and CD8 T-cells toxicity and inflammatory response were also predicted to be significantly activated in CKD4 in comparison to the CKD1 group. These observations suggest the presence of variable cytotoxic gut inflammation in CKD4 patients, which negatively affects the epithelial gut lining, resulting in gradual deterioration and increased permeability. Even more, we identified cell growth and carbohydrate degradation and synthesis pathways, important for energy production, to be significantly enriched in CKD4 in comparison to the CKD1 group. The activated multiple cell growth pathways are a potential coping mechanism to maintain epithelial integrity, among other cellular attempts (enriched carbohydrate synthesis) to achieve restoration and remodeling of the damaged tissues.

Along these lines, Intestinal-type alkaline phosphatase (*ALPI*) was found to be decreased in abundance in the fecal samples from patients with CKD4 versus CKD1. *ALPI* is a brush border enzyme secreted by the intestinal enterocytes and is involved in the absorption of fatty acids. It is a known anti-inflammatory gut enzyme that dephosphorylates lipopolysaccharides (LPS), hence, maintaining intestinal microbial homeostasis and epithelial barrier function [38]. Deficiency of *ALPI* resulting from a loss in expression or damage to the protein, has been reportedly linked to diabetes, obesity, chronic intestinal inflammation, gut dysbiosis, bacterial translocation and systemic inflammation, while it is further associated with DNA damage and oxidative stress [39,40], in line with the present results. In parallel, Galectin-3-binding protein (*LGALS3BP*) was identified to be up-regulated in CKD4 versus CKD1. *LGALS3BP* is a secreted glycoprotein involved in promoting integrin-mediated cell-adhesion and immunomodulation [41]. Furthermore, a decrease in *LGALS3BP* has been associated with a reduction of colon inflammation in a study performed in mice, suggesting its role as a potential immunotherapeutic target for colon inflammation [42]. We may therefore hypothesize that its upregulation in CKD4 may reflect increased inflammation occurring at this disease stage. Decreased levels of Phospholipase A2 (*PLA2*) were identified in CKD4 versus CKD1. *PLA2* plays a role in the biosynthesis of N-acyl ethanolamines that regulates energy metabolism and inflammation in the intestinal tract [43]. Prolactin-inducible protein (*PIP*), identified only in the CKD4 group in the present study, is a glycoprotein involved in the immune response and possesses the potential to inhibit bacterial species growth [44]. Increased levels of this protein in CKD4/CKD1 may be due to the previously documented elevated prolactin (considered a uremic toxin) levels in CKD [45].

Prostaglandin G/H synthase 1 (*PTGS1*) was identified only in the CKD4 group. *PTGS1* is involved in the constitutive production of prostanoids, which are important for inflammatory response, in different organs. For instance, in gastric epithelial cells, *PTGS1* is vital for the generation of prostaglandin E2, important in cyto-protection [46]. Additionally, enzymes taking part in the food digestion pathways, predominantly in the intestine, were also identified to be differentially abundant in the present study, including Very long-chain specific acyl-CoA dehydrogenase, mitochondrial (*ACADVL*) which was expressed only in CKD4 patients. ACADVL supposedly initiates the anaerobic breakdown of fatty acids [47]. The other digestive enzymes, Pancreatic alpha-amylase (*AMY2A*) and Alpha-amylase 2B (*AMY2B*), were down-regulated in CKD4 versus CKD1. No studies directly linking these digestive enzymes to inflammation and/or CKD could be found in the literature. In general, *AMY2A* and *AMY2B* are known to regulate/control blood sugar during intestinal absorption, by initiating starch digestion. At high glucose concentrations, they can also inhibit the uptake of glucose by Na+/glucose cotransporter 1 (*SGLT1*) [48]. Hence, the down-regulation of *AMY2A* and *AMY2B* proteins as observed in CKD4 in comparison to the CKD1 group, potentially implies an increased glucose uptake in the CKD4 group, leading to a glucose imbalance, and a reduced digestion of starch, which may contribute to reduced saccharolytic fermentation, crucial for the synthesis of SCFAs such as butyrate. This hypothesis is supported by the quantitative ELISA results (Fig. 4) depicting a trend of decrease in *AMY2A* concentration in CKD4 fecal suspension samples compared to CKD1, with a fold change ratio of 0.42. We may therefore hypothesize that butyrate synthesis pathways are affected in more advanced stages of CKD, a result which was further supported by the bacterial proteomic data, as described below.

The bacterial species in the fecal suspension samples of CKD patients generating the highest number of proteins was *E. coli* (as expected [49]), but also *Bacteroides fragilis*, *Bacteroides thetaiotaomicron* and *Phocaeicola vulgatus*. *B. fragilis*, an anaerobic gram-negative species residing in lower intestine of humans, is widely associated with inflammatory diseases. *B. fragilis* produces polysaccharide A and SCFA, stimulating IL-10 production of CD4 T-regulatory cells, plausibly inhibiting inflammation. *B. thetaiotaomicron* ferments dietary fructans to yield acetate. The capacity of *B. thetaiotaomicron* to generate the protein-bound uremic toxin precursor indole and indole-3-acetic acid (IAA) has been described in the literature [50,51]. The strain *P. vulgatus* (earlier *Bacteroides vulgatus* [52]) is one of the highly abundant *Bacteroides* members of the human gut microbiota. Liu et al. [53], highlighted that the effect of *P. vulgatus* on inflammation is strain-specific and reported the protective effect of *P. vulgatus* Bv46 strain in moderating the symptoms of colitis, intestinal microbiota and intestinal immune response. In the present study, bacterial proteins identified from the strain *P. vulgatus* ATCC 8482/DSM 1447, reportedly possess a potential probiotic activity in the intestines [54]. The *P. vulgatus*, *B. fragilis* and *B. thetaiotaomicron* strains together are predominantly known to be responsible for reducing the intestinal inflammatory response.

Along the same lines, eight out of the nine significantly differentially abundant bacterial proteins participate in essential pathways responsible for host energy homeostasis, including degradation and synthesis of carbohydrates through Kreb's cycle, glycolysis, gluconeogenesis, and pentose phosphate pathway, taking place under aerobic and anaerobic conditions. These results are in line with a metaproteomic study that analysed fecal samples from eight infants, which reported abundantly expressed proteins associated with carbohydrate, energy, and amino acid metabolism and Kreb's cycle [36]. Interestingly, the products of these pathways: glutamate, succinate and acetyl-CoA are precursors in the synthesis of SCFAs such as butyrate [55]. The differential abundance of these energy homeostasis proteins identified in CKD4 in comparison to the CKD1 group, combined with their importance in the synthesis of SCFAs, further supports the hypothesis that butyrate synthesis might be affected with CKD progression. Interestingly, in a previous study performed on the fecal suspension samples from the same cohort [25], a statistically significant positive correlation between the concentrations of butyric acid and eGFR was observed. Specifically, quantification of butyrate in the fecal suspension samples used in the present study, showed a decreasing trend in butyrate levels from CKD1 (15.65 ± 8.84 μmol/g feces) to CKD4 (12.01 ± 8.59 μmol/g feces) with a fold change of 0.77 (Table 1 and Table S1; no statistical significance could be achieved plausibly due to the low sample size), suggesting the decrease in available fecal butyrate levels with deteriorating kidney function [25]. The differential abundance of bacterial proteins has similarly been linked to the aberrant synthesis of butyrate in other fecal metaproteomic studies on inflammatory bowel syndrome or Crohn's disease [37]. Additionally, the bacterial protein Glutaredoxin 1 (*grxA*) in the present study, shown to be down-regulated in CKD4 in comparison to CKD1, is a thiol-transferase enzyme, primarily responsible for removal of protein glutathionylation. Inactivation of *grxA* may induce the aberrant glutathionylation of proteins [56] which has been linked to disturbance of cell function and normal redox signalling, potentially associated with oxidative stress and kidney damage in CKD. Research on effects of reducing glutathionylation in 90 diabetic CKD patients and 45 healthy subjects, demonstrated its beneficial role in reversing oxidative stress and protecting against kidney damage, promoting its use as a potential therapeutic target in CKD treatment [57]. Interestingly, *grxA* was detected from the highly abundant bacterial species *Escherichia coli*, arising the hypothesis of potential respective reduced gene expression.

The present study has a set of limitations, namely the small sample size and the difference in average age of the participants from the two CKD groups, as CKD is a chronic and progressive disease, matching for age between the two extreme disease stages is highly challenging. Additionally, the absence of an easily accessible and reproducible human-gut-microbiome database for protein identification, and the lack of bioinformatic tools available for integrating the human and bacterial proteins, are limitations that may also partly explain why metaproteomic analyses are still lagging behind the metagenomics studies. Hence, the present study underscores the need for expansion with more datasets from multiple molecular levels to increase the power of observations, comprehensiveness, and coverage along with validation of the observed changes in CKD. Metagenomics and metaproteomics data integration from the same cohort with the aim to explain the link between the genetic capacity of the gut microbiota and the fecal end-products in uremic conditions is planned.

## 5 Conclusions

• This study provides a source of research data supporting that fecal samples are a highly relevant biological material reflecting interactions along the host-gut-kidney axis.
• The data highlighted differences in abundance of human proteins, associated with inflammation and known molecular changes in CKD, serving as positive control for this exploratory metaproteomics analysis.
• Furthermore, observed changes in human proteins such as *AMY2A* suggested an increased glucose uptake in advanced kidney disease (for example, when comparing CKD4 to CKD1), resulting in potential glucose imbalance and reduced digestion of starch; this further suggests reduced saccharolytic fermentation, crucial for the synthesis of SCFAs.
• Bacterial proteins with differential abundance in CKD4 versus CKD1 were found to be associated with essential carbohydrate and energy metabolism, that interestingly produce the precursors of SCFAs such as butyrate.
• The verified reduced levels of fecal butyrate and *AMY2A* in CKD4 in comparison to CKD1, support the hypothesis that butyrate synthesis pathways are affected with CKD progression and these changes, may be reflective of changes in the gut barrier integrity.

## Ethical statement

The study was conducted in accordance with the Declaration of Helsinki and approved by the Medical Ethics Committee of Gent University Hospital (Ref. No. 2010/033, B67020107926) on 06-10-2020. Written informed consent was obtained from all the 29 volunteers.

## Data availability statement

The LC-MS/MS proteomics data have been deposited to the ProteomeXchange Consortium via the PRIDE partner repository, with the dataset identifier PXD048778 and DOI 10.6019/PXD048778. reviewer_pcd048778@ebi.ac.ukThis paper does not report original code. Any additional information required to reanalyze the data reported in this paper is available from the corresponding authors upon request.

## Funding

This project has received funding from the European Union's 10.13039/501100007601Horizon 2020 research and innovation programme under the Marie Skłodowska-Curie Actions (MSCA) grant agreement No 860329.

## CRediT authorship contribution statement

**Sonnal Lohia:** Writing – original draft, Visualization, Software, Methodology, Investigation, Formal analysis, Data curation, Conceptualization. **Sophie Valkenburg:** Writing – review & editing, Validation, Conceptualization. **Rafael Stroggilos:** Writing – review & editing, Software, Data curation. **Vasiliki Lygirou:** Methodology, Formal analysis. **Manousos Makridakis:** Software, Methodology, Formal analysis. **Jerome Zoidakis:** Validation, Supervision, Resources. **Francis Verbeke:** Writing – review & editing, Supervision. **Griet Glorieux:** Writing – review & editing, Supervision, Resources, Project administration, Funding acquisition. **Antonia Vlahou:** Writing – review & editing, Supervision, Resources, Project administration, Funding acquisition, Conceptualization.

## Declaration of competing interest

The authors report that there are no competing interests to declare. Some components of the study design figure (Fig. 1) were exported from biorender.com.

## Figures and Tables

**figs1:**
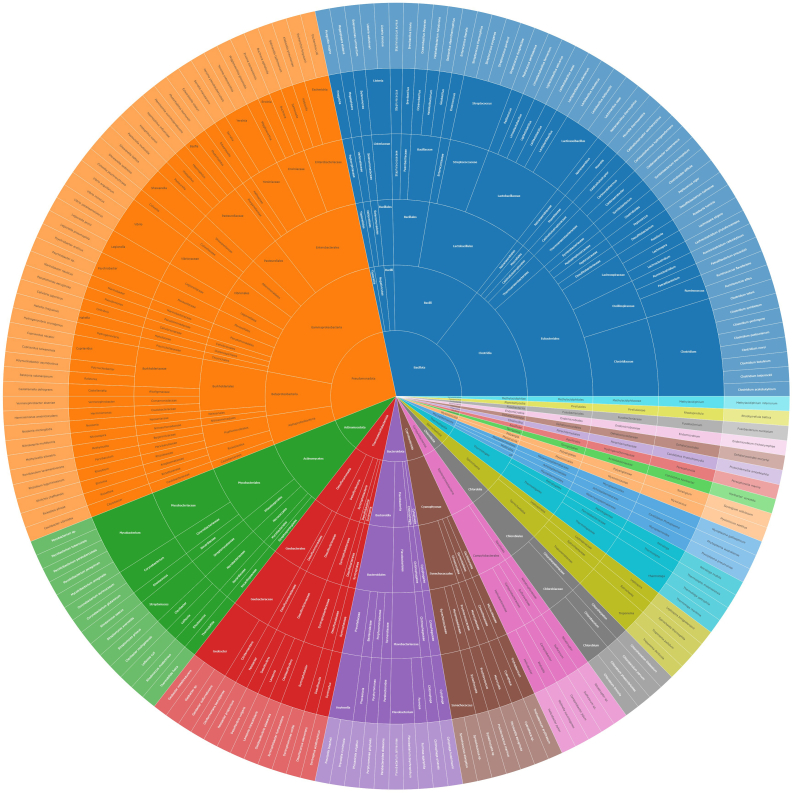
Sunburst chart representating the taxonomical heirarachy of the bacterial species from which bacterial proteins were identified in CKD1.

**figs2:**
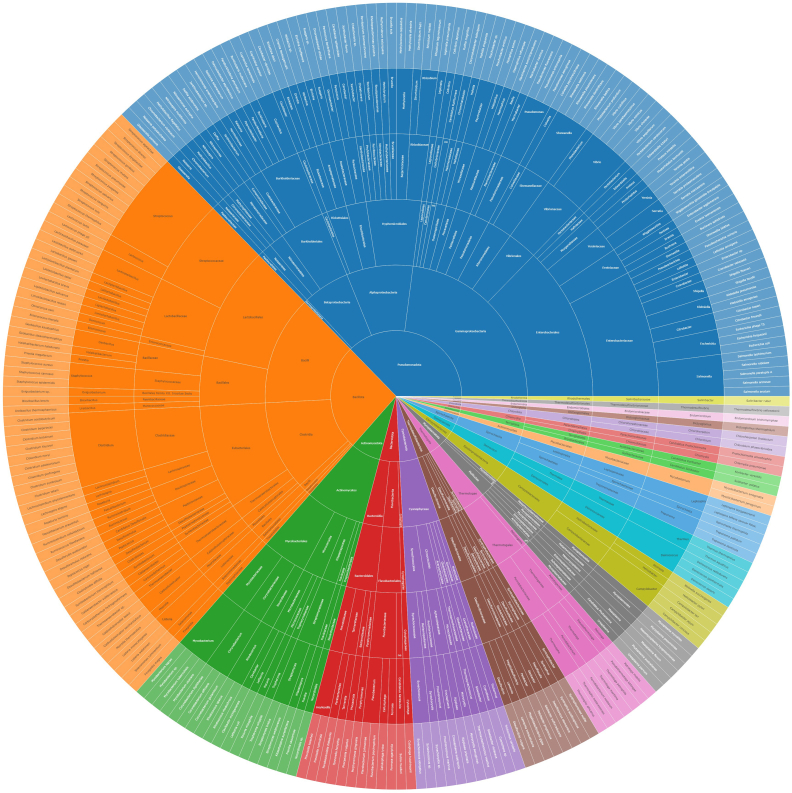
Sunburst chart representating the taxonomical heirarachy of the bacterial species from which bacterial proteins were identified in CKD4.
