# Supplementary material for: Investigation of the human-gut-kidney axis by fecal proteomics, highlights molecular mechanisms affected in CKD
Source: Heliyon. 2024 Jun 11;10(12):e32828. doi: 10.1016/j.heliyon.2024.e32828 (PMC11226915; doi:10.1016/j.heliyon.2024.e32828)
Supplement: Multimedia component 9 [file mmc9.docx]

**Index for Supplementary Information**

**Tables**

**Table S1**: Patient characteristics

**Table S2**: Proteins identified from the Human database of UniProtKB

**Table S3**: Pathway enrichment scores obtained from ssGSEA analysis linked to the proteins identified from the Human database

**Table S4**: Results of ELISA performed for *AMY2A* (Pancreatic alpha-amylase; P04746) protein in fecal suspension samples

**Table S5**: Proteins identified from the Bacterial database of UniProtKB

**Table S6**: KEGG pathways associated with proteins identified from the top four bacterial species generating highest number of proteins

**Table S7**: Gene ontology analysis for the statistically significant bacterial proteins

**Table S8**: Proteins participating in the Butyrate synthesis pathway, that were identified only in one CKD group

**Supplementary Document: Pipeline for identification of proteins**

**2.1. Study population:**

- Fecal samples were collected from patients with CKD stage 1 (CKD1, *n* = 12) and CKD stage 4 (CKD4, *n* = 17), recruited at the Nephrology Unit of the Gent University Hospital, Belgium, as previously described.
- Exclusion criteria that were applied include: dialysis treatment, active infection (C-reactive protein (CRP) > 20 mg/L), immunosuppressive therapy, body mass index (BMI) > 35 kg/m^2^, inflammatory bowel disease, active malignancy, cardiovascular event in the past 3 months, pregnancy, transplantation, use of non-steroidal anti-inflammatory drugs within the past month, and age < 18 years.

**2.2. Sample preparation:**

- Fecal suspensions were prepared from the fecal samples. Briefly, 5 mL of anaerobic phosphate buffer was added per 1 g of the feces in a 15 mL falcon tube and vortexed for 10 minutes. The fecal suspensions obtained were filtered with a 0.22 µm filter to remove viable bacteria and stored in aliquots of 500-1000 µL at -80°C, until further processing.
- Samples were prepared for high-throughput proteomic analysis with the GeLC-MS protocol.
- Next, trypsinization was performed with the addition of 600 ng trypsin in 10 mM ammonium bicarbonate pH 8.5, per sample, followed by incubation at room temperature in the dark for 12-16 hours under humidified conditions.
- Finally, the extracted tryptic peptides were cleaned with 0.22 µm PVDF filters, dried with a centrifugal vacuum concentrator and stored at −80 °C until LC-MS/MS analysis.

**2.3. LC-MS/MS analysis:**

- The instrument used for the analysis was a Dionex Ultimate 3000 RSLC nanoflow system (Dionex, Camberley, United Kingdom) coupled with the high-resolution QExactive Orbitrap mass spectrometer (Thermo Fisher Scientific, Bremen, Germany).
- MS1 ion resolution was set to 70,000 and that for high-energy collisional dissociation at MS2 was set to 35,000. MS/MS mode scanning was set from 380 to 2,000 m/z.
- Applying 35% collision energy of HCD, selection of top 20 multiply charged ions from each MS/MS scan was performed.
- Targeted precursors with 5 ppm mass tolerance were dynamically excluded for 30 seconds for further isolation and activation.

**2.4. Protein identification:**

- Raw files (.raw) obtained from the LC-MS/MS instrumental set up were analysed by Proteome Discoverer 1.4 software with the SEQUEST search engine against the entire reviewed non-redundant human (accessed on 03-01-2023; https://www.uniprot.org/ > UniProtKB Keyword search: Human > Reviewed) and bacterial (accessed on 03-01-2023; https://www.uniprot.org/ > UniProtKB Keyword search: Bacteria > Reviewed) databases downloaded as FASTA files.
- Cysteine carbamidomethylation and methionine oxidation were selected as fixed and dynamic modifications, respectively.
- The digestive enzyme was selected as trypsin.
- During the search, a precursor mass tolerance of 10 ppm, two missed cleavage sites, peptide length of 6–144 amino acids, and 0.05 Da fragment mass tolerance were permitted, with the false discovery rate (FDR) set stringently at 0.01, yielding a high confidence output.

**2.5. ELISA:**

- The quantification of Pancreatic alpha-amylase (*AMY2A*; P04746) protein (56 kDa) in fecal suspension samples was performed by enzyme-linked immunosorbent assay (ELISA) using the Pancreatic Amylase Human ELISA Kit (#ab137969, Abcam, Cambridge, UK) as per the manufacturer’s instructions, using 50 µL of diluted sample per well.

**2.6. Statistical analysis:**

- The precursor ion area values as defined by Proteome Discoverer 1.4, was merged using an in-house R script and were subjected to a global (per sample) normalization as per the formula: *X*′ = (*X*/sum(*Xi*)) * 10^6^; where *X* is the raw protein area; sum(*Xi*) is the sum of all raw protein areas of a given sample; and *X′* is the normalized protein area.
- The non-parametric Mann-Whitney *U* test was utilized for defining statistical significance. The *p*-values below 0.05 were considered as statistically significant.

Note: In the search with the bacterial database, a total of 3058 proteins were identified from the 29 fecal suspension samples. After excluding the duplicates (*n* = 1721; protein entries that were repeated due to identification in more than one fecal suspension sample), proteins of human origin (*n* = 281) and additional contaminants (*n* = 45 proteins of non-bacterial origin including sheep, fish, fungi, amoeba amongst others present in the downloaded UniProtKB database FASTA), a total of manually cross-checked, 1011 proteins of bacterial origin, were identified in this analysis (Table S5).

**2.7. Bioinformatic analysis:**

**Human database**

- For further biological insights into the proteomic profiles obtained from the human database, single-sample gene set enrichment analysis (ssGSEA) was conducted using the gene set variation analysis (GSVA) algorithm in R from the Bioconductor package (version 1.32.0).
- The GSVA-ssGSEA method calculates the activity scores for each pathway in each sample.
- Pathways used in the GSVA analysis were extracted from the MSigDB database (https://www.gsea-msigdb.org/gsea/msigdb/index.jsp, version 7.2).
- We specifically utilized the following collections: H – hallmark gene sets (*n* = 50), C2 – curated gene sets (*n* = 6495), C3:TFT – regulatory gene sets:all transcription factor targets (*n* = 1115), C5:GO – ontology gene sets:gene ontology (*n* = 10532), C7:IMMUNSIGDB – immunologic gene sets:ImmuneSigDB (*n* = 4872), and C8 – cell type signature gene sets (*n* = 830).
- The data was z-score normalised per row (per protein).
- Pathway activation scores were compared among the two CKD groups with the Mann-Whitney *U* test, and significance was defined at *p*-value < 0.05 and direction of over-activation for a pathway was determined with the fold difference of the means (= Mean activation score in CKD4 – Mean activation score in CKD1).

**Bacterial database**

- For insights into the biological role of the proteins identified from the statistically significant bacterial database, Gene Ontology (GO) Term IDs were retrieved from their respective protein accession IDs using UniProtKB (https://www.uniprot.org/id-mapping).
- The collected GO Term IDs were inputted into the open-source online tool REVIGO (http://revigo.irb.hr/), which uses semantic similarity measures to convert long lists of GO terms to summarized molecular functions, cellular components, and biological processes.
- Furthermore, bacterial species generating 10 or more proteins were short-listed, and the top four species were determined by the highest number of identified proteins originating from the respective bacterial species.
- The protein interaction network and pathway enrichment analysis were performed using the online search tool for protein-protein interaction (PPI) networks, STRING database (https://string-db.org/, version 11) at the default settings, for all the proteins mapped to the top four bacterial species.
